# PATHWAYS TO A GOOD LIFE? MULTIPLE SOCIAL ROLES IN ADULTHOOD AND MENTAL WELL-BEING IN LATER LIFE IN EUROPE

**DOI:** 10.1093/geroni/igz038.3339

**Published:** 2019-11-08

**Authors:** Christian Deindl, Miriam Engels

**Affiliations:** 1 Heinrich-Heine-University Düsseldorf, Centre for Health and Society, Düsseldorf, Nordrhein-Westfalen, Germany; 2 Heinrich-Heine-University Düsseldorf, Centre for Health and Society, Düsseldorf, Germany

## Abstract

The connection between employment, family life and health is well documented. Job demands and family obligations are divergent responsibilities and can be a constant source of conflict. The resulting role strain can have a long lasting impact on mental health. Using data from SHARE and ELSA, we take a life course perspective and look at patterns of employment history from the age of 25 to 40 combined with partnership and fertility history of 17,189 men and 23,266 women in 22 European countries. Sequence analysis combined with cluster analysis shows a clear picture of five dominant states in our sample: Stable work and family, stable work without family, working single parent, working childless couples, and being non employed. This pattern is similar for men and women. We use path models to distinguish the impact of childhood conditions on such life course patterns and the direct and indirect impact of employment and family life on mental health. Women who did not combine work and family roles, (work without family, family without work) reported higher levels of depression in comparison with women who combined work and family. Non-working women and single mothers also experienced indirect effects on depression through their economic situation. Unemployed men or men without family reported higher levels of depression. Unemployment and being a single father also have an indirect impact on depression via economic conditions and health. Moreover, such results also differ between countries, with lower employment rates reducing role strain for women, but not so for men.

